# SCOWLP classification: Structural comparison and analysis of protein binding regions

**DOI:** 10.1186/1471-2105-9-9

**Published:** 2008-01-08

**Authors:** Joan Teyra, Maciej Paszkowski-Rogacz, Gerd Anders, M Teresa Pisabarro

**Affiliations:** 1 Structural Bioinformatics, BIOTEC TU-Dresden, Tatzberg 47-51, 01307 Dresden, Germany; 2 Max Planck Institute for Molecular Cell Biology and Genetics, Pfotenhauerstrasse 108, D-01307 Dresden, Germany

## Abstract

**Background:**

Detailed information about protein interactions is critical for our understanding of the principles governing protein recognition mechanisms. The structures of many proteins have been experimentally determined in complex with different ligands bound either in the same or different binding regions. Thus, the structural interactome requires the development of tools to classify protein binding regions. A proper classification may provide a general view of the regions that a protein uses to bind others and also facilitate a detailed comparative analysis of the interacting information for specific protein binding regions at atomic level. Such classification might be of potential use for deciphering protein interaction networks, understanding protein function, rational engineering and design.

**Description:**

Protein binding regions (PBRs) might be ideally described as well-defined separated regions that share no interacting residues one another. However, PBRs are often irregular, discontinuous and can share a wide range of interacting residues among them. The criteria to define an individual binding region can be often arbitrary and may differ from other binding regions within a protein family. Therefore, the rational behind protein interface classification should aim to fulfil the requirements of the analysis to be performed.

We extract detailed interaction information of protein domains, peptides and interfacial solvent from the SCOWLP database and we classify the PBRs of each domain family. For this purpose, we define a similarity index based on the overlapping of interacting residues mapped in pair-wise structural alignments. We perform our classification with agglomerative hierarchical clustering using the complete-linkage method. Our classification is calculated at different similarity cut-offs to allow flexibility in the analysis of PBRs, feature especially interesting for those protein families with conflictive binding regions.

The hierarchical classification of PBRs is implemented into the SCOWLP database and extends the SCOP classification with three additional family sub-levels: Binding Region, Interface and Contacting Domains. SCOWLP contains 9,334 binding regions distributed within 2,561 families. In 65% of the cases we observe families containing more than one binding region. Besides, 22% of the regions are forming complex with more than one different protein family.

**Conclusion:**

The current SCOWLP classification and its web application represent a framework for the study of protein interfaces and comparative analysis of protein family binding regions. This comparison can be performed at atomic level and allows the user to study interactome conservation and variability. The new SCOWLP classification may be of great utility for reconstruction of protein complexes, understanding protein networks and ligand design. SCOWLP will be updated with every SCOP release. The web application is available at .

## Background

Protein interactions are essential for intra-cellular communication in biological processes. Proteins are composed of small units or domains that can physically interact together forming multi-domain protein complexes. A single protein can have several binding regions, and each region can engage distinct ligands, either simultaneously or at successive stages of signalling [1].

In our previous work we developed the SCOWLP database [2], which contains detailed interfacial information of structurally known protein complexes, peptide complexes and water molecules as mediators of interactions. SCOWLP and other existing protein interaction databases [3-5] contain lists of interfaces for SCOP protein families and, therefore, they are only able to perform individual interface analysis. A classification of protein binding regions (PBRs) is essential in order to characterize all protein regions participating in the binding and to be able to compare protein complexes sharing the same binding region. At the same time, such a classification should provide some insights into the interacting properties preserved by members of a protein family. However, the criteria to delineate PBRs can be difficult to assess, and often arbitrary and conflictive.

Binding regions in protein domains can form separated patches, but also some protein families bind through multiple binding regions with different ranges of residue overlapping. Furthermore, some observed protein interfaces are the result of non-biological artefacts (i.e. crystal packing) and are often difficult to distinguish from the biological ones, creating discrepancy among the current resources [6,7]. Some of these interfaces can connect binding regions or can be included into existing ones, introducing noise quite difficult to handle for clustering algorithms. As different clustering algorithms can vary the grouping completely, an advantageous classification of PBRs should contain a proper measurement of similarity and a flexible clustering algorithm to cover the requirements of the analysis to be performed.

Hierarchical clustering comprises a whole family of clustering methods differing only on the manner inter-cluster distance is defined (the linkage function). The more common aggregation methods are single-, complete- and average-linkage. Complete and single-linkage are extreme procedures with completely different properties. Complete-linkage uses the similarity between the furthest pair of objects from two clusters. In contrast to these requirements, single-linkage only uses the nearest pair of objects from each cluster. Both methods have an extreme conception of homogeneity of a cluster. Single-linkage leads to grouping and may result in a few large and heterogeneous clusters [8]. Complete-linkage results in dilatation and may produce many clusters, being more suitable for isolating poorly separated clusters [9]. Average-linkage tries to avoid these effects by computing the average. This method is used by two different computational approaches for protein interface classification described so far. Nussinov and colleagues pioneered interface classification based on common structural features shared among the interfaces from various folds and considering full interfaces at chain level [10-12]. More recently Kim and colleagues [13,14], instead of classifying interfaces as a whole, classified the domain faces forming an interface by SCOP families. Their classification uses interfaces defined as biological in the PQS database [6] and does not include peptidic and solvent interaction data.

We present a classification of PBRs from all existing contacting domains from the SCOWLP database, which includes detailed information about proteins, peptides and solvent interaction. Peptide and solvent interactions are highly represented in the PDB and are highly informative in protein interactions [15]. For our classification we use hierarchical clustering with the complete-linkage method. The similarity measure used is obtained based on the overlapping of interacting residues mapped in pair-wise structural alignments and exclusion of gap regions. We explain and discuss the methodology used to classify PBRs and the rational behind applying flexible similarity cut-offs. Our PBRs classification is implemented in SCOWLP and extends its usage from individual analysis of protein interfaces to comparative structural analysis of specific family binding regions. We describe the SCOWLP web application and its utilities for PBRs analysis.

## Construction and content

**Figure 1:**
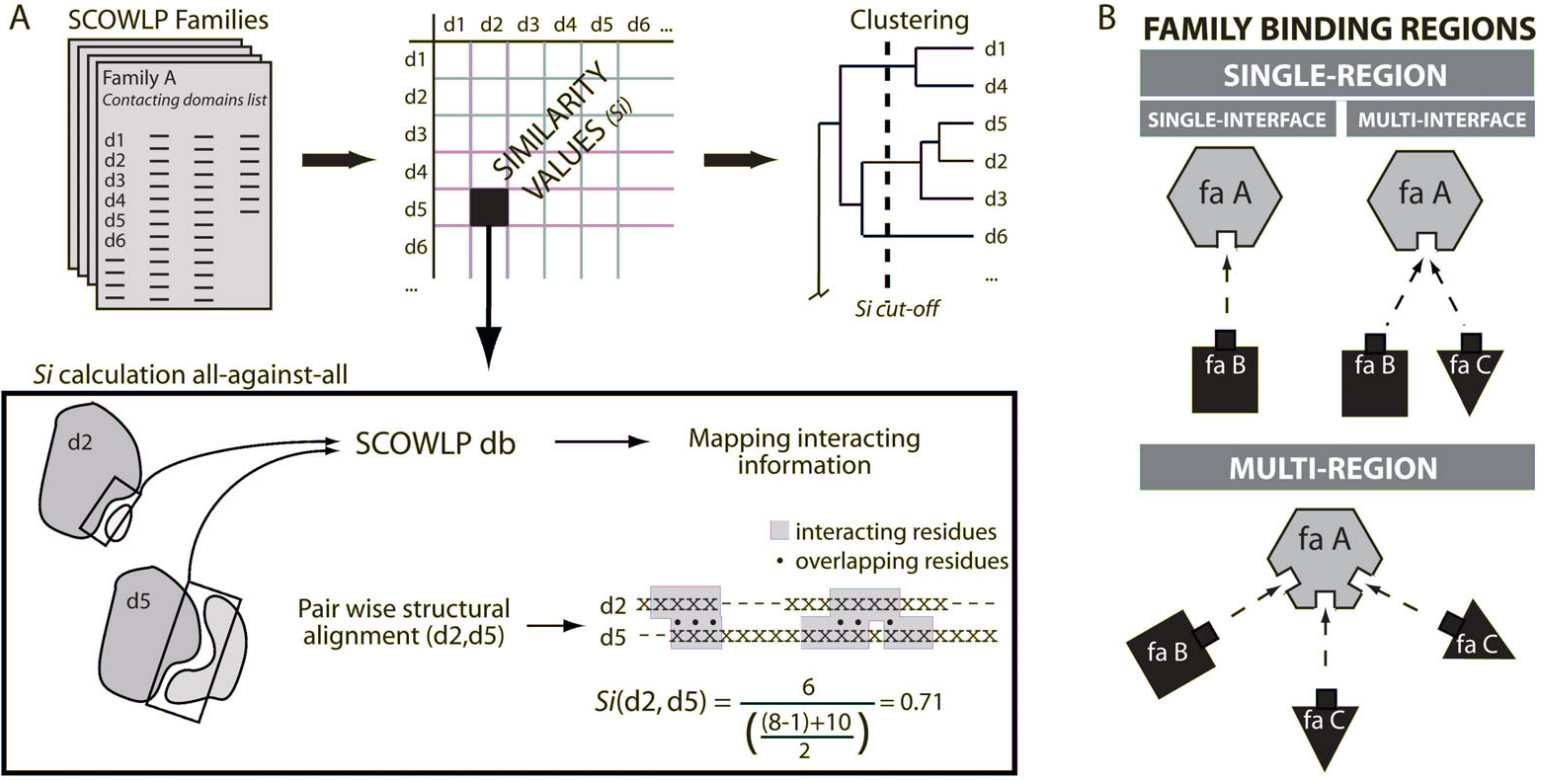
**Schematic overview of the methodology**. A) The contacting domains per family are extracted from the SCOWLP database. The *Si *are calculated for all-against-all contacting domains and used for clustering. Results are displayed in a dendrogram. B) Classification of protein binding regions (PBRs). A protein family can recognize other proteins and ligands by single- or multi-binding regions, and for each binding region single- or multi-interfaces may exist depending on the number of partners that have been structurally observed interacting within a specific binding region.

The PBRs classification extends the SCOWLP relational database by four additional tables describing the hierarchical classification at binding region, interface and contacting domain level. The content of the classification is given at similarity zero, which offers a general view of the regions that protein families use for recognition. In addition, our classification offers different similarity cut-offs to allow flexibility in the analysis of the PBRs. The classification of PBRs was performed as follows (Fig. 1A):

### 1 Extraction of interfaces and contacting domains

An accurate definition of the interacting residues is crucial to have a proper clustering of a family PBR. We extracted all protein interfaces from the SCOWLP database, in which the interactions are defined at atomic level and based on their physiochemical properties [2,15]. Protein domains interacting with peptidic ligands and residues interacting through a water molecule (*wet spots*) are also taken into account. We consider "interface" all domain-domain interactions; that means those belonging to the same protein and also to different proteins. SCOWLP contains 79,803 interfaces contained in 2,561 SCOP families. We grouped the domains participating in each interface by SCOP families, obtaining for each family a list of contacting domains with the residues forming part of the binding region.

### 2 Pair-wise structural alignments (PSAs)

A reliable alignment is indispensable to calculate the similarities among binding regions. For this purpose we used MAMMOTH, which has shown proven accuracy to structurally align protein families [16]. We performed all-against-all PSAs of the contacting domains for each family to be able to measure the similarity among binding regions. SCOWLP contains about 160,000 contacting domains uneven distributed by families. This represents 276 million PSAs performed in a cluster of five Pentium IV 2.6 GHz. The alignments were performed taking the Cα atoms into account and using a gap penalty function for opening and extension [17]. The root-mean-squared deviation (RMSD) was not considered for measuring the similarity between two interfaces, as the superimposed members of the same family share a common structure.

### 3 Similarity Index (*Si*)

The residues described in SCOWLP to be forming and interface were mapped onto the domain-pair structural alignment. We calculated a similarity index (*Si*) based on the number of interacting residues that overlap and the length of both interacting regions by (Fig. 1):



where *a *and *b *represent the two domain structures aligned. The number of interacting residues that match in the PSA is represented by *IR*_*overlap*(*a*, *b*)_. This value is divided by the average number of the interacting residues in both domains excluding the interacting residues located in gap regions in the structural alignment (*IR*_*gaps*_).

### 4 Clustering binding regions

Based on the calculated *Si*, we clustered the binding regions of each SCOP family using the agglomerative hierarchical algorithm [8] following several steps (Fig. 1):

1) Define as a cluster each contacting domain.

2) Find the closest pair of clusters and merge them into a single cluster.

3) Re-compute the distances between the new cluster and each of the remaining clusters.

4) Repeat steps 2 and 3 until all contacting domains are clustered into a single cluster.

To re-compute the distances we used the complete-linkage method [9], which considers the distance between two clusters to be equal to the minimum similarity of the two members.

### 5 Binding region definition by *Si *cut-offs

**Figure 2:**
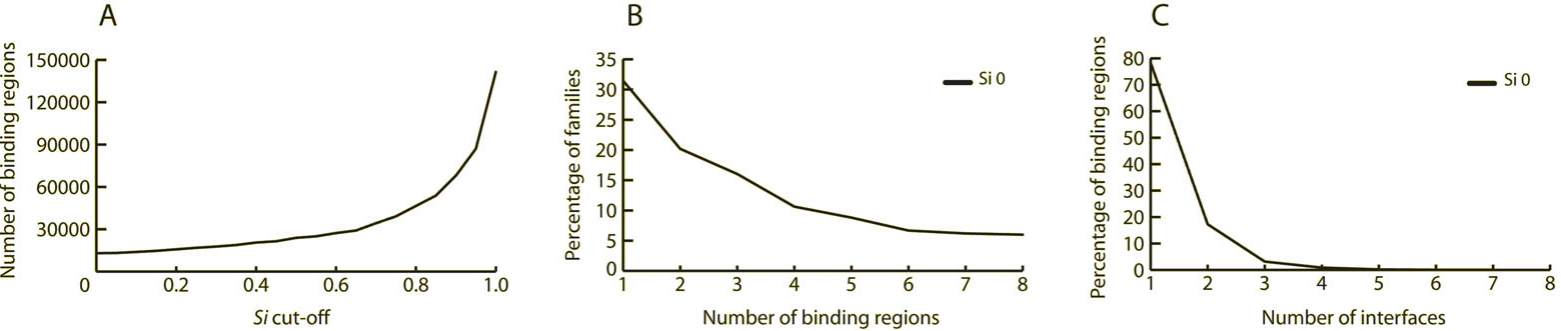
**PBR analysis**. A) Representation of the number of binding regions obtained using different *Si *cut-offs. B) Representation of the relative percentage of SCOP families with different number of binding regions at zero *Si *cut-off. C) Representation of the relative percentage of binding regions depending on the number of interfaces at zero *Si *cut-off. (x-axis in B and C are limited to 8 for simplicity).

The result of the clustering can be represented in an intuitive tree or dendrogram, which shows how the individual contacting domains are successively merged at greater distances into larger and fewer clusters. The final PBRs depend on the *Si *cut-off that is set up. We can observe in Fig. 2A that the total number of binding regions for all the SCOP families grows exponentially as the *Si *cut-off increases. Based on our observations of a representative group of families we set up an empirical maximum similarity cut-off value of 0.4. We pre-calculated the results for *Si *cut-offs at 0, 0.1, 0.2, 0.3 and 0.4 to offer a range of values that allow flexibility in the final analysis of PBRs. The SCOWLP web application offers the possibility to display the classification at any of these cut-off values.

Our classification clustered 160,000 contacting domains from 2,561 families in 9,334 binding regions. About 65% of the families contain more than one binding region (Fig. 2B). These values are obtained for similarity zero and may vary depending on the similarity cut-off applied.

### 6 Interface definitions

In order to differentiate binding regions having single-interfaces from multi-interfaces (Fig. 1B), we identified in each binding region the partner for each contacting domain. Each binding region was divided in sub-clusters when there were different domain families interacting in the same binding region. This resulted in a total of 10,300 interfaces. The classification shows a 78% of the binding regions having a single-interface and the rest having mainly 2 or 3 interfaces per region (Fig. 2C). These numbers have to be carefully interpreted by taking into account the limitation of the structural information contained in the PDB (*i.e. *1,715 binding regions contain a unique member in the PDB and therefore only one known interface per binding region).

### Implementation

We used MySQL and Java programming language to generate the classification of PBRs. Calculations were performed on a cluster of five Pentium IV 2.6 GHz. The PBRs classification has been included into the SCOWLP database. SCOWLP will be updated with every SCOP release.

## Utility and Discussion

In this section we first discuss the methodology used for the classification of PBRs. Besides, we describe the utility of the SCOWLP web application.

### Extraction of similarities

The classification of PBRs requires a proper definition of the similarity between binding regions. For this purpose it is essential to have (a) a reliable source of interface definitions, (b) high quality alignments, and (c) a adequate similarity function:

#### a) Interface definitions

Our work includes detailed atomic interfacial information from the SCOWLP database, which comprises protein-protein complexes, protein-peptides complexes and solvent-mediated interactions [2,15]. The contained interacting information at physicochemical level is very useful to study and compare conservation/variability among complexes even at low sequence similarity.

#### b) Domain PSAs of a family are computationally efficient and give reliable Si

The two partners forming an interface do not have to be aligned in order to extract a *Si*. For each interface, only the partner belonging to the family in study is structurally aligned with the rest of the members of the family. This procedure has two clear advantages: (1) an increased computational speed for each PSA as we overlook one of the partners, reducing the amount of residues to align, (2) the good quality of the family domain alignments, as family domains are structurally conserved. Protein binding regions are often irregular, discontinuous and difficult to compare. Therefore, a good alignment is critical to calculate the range of overlap between two regions. Our classification method is exclusively based on structural alignments, which makes the methodology computationally expensive but gives better accuracy than sequence alignments at family level.

#### c) The similarity index penalizes gap regions

The *Si *reflects the overlap of interacting residues between two binding regions in domains belonging to the same protein family. It is important to consider the number of interacting residues per domain, which allows us to obtain the percentage of interacting residues that is overlapping over the total (see Methods). This helps to distinguish whether a binding region is identical, different or included into another one.

**Figure 3:**
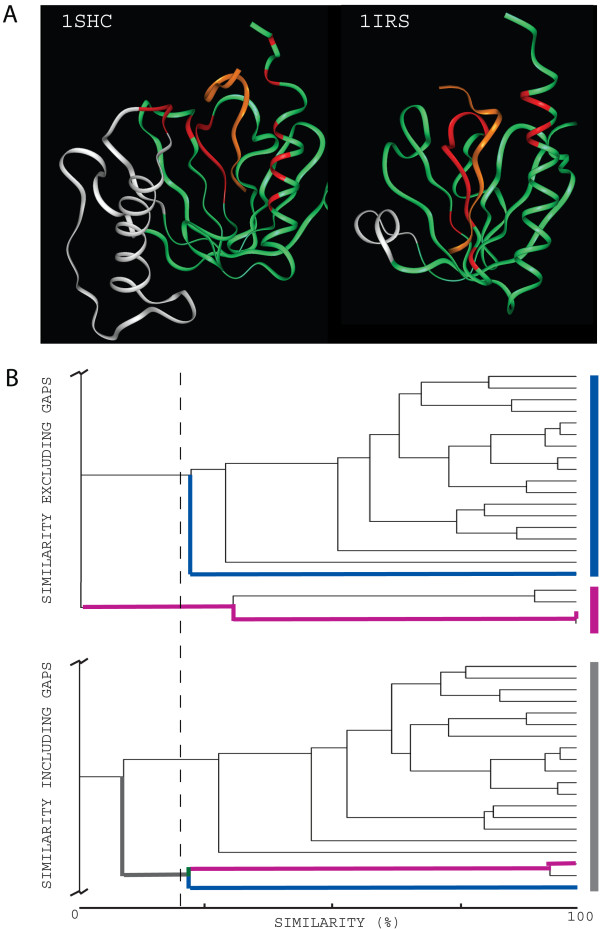
**Effects of gap regions for *Si *calculation and clustering**. A) Two structures of the PTB domain differing in an insertion/deletion are displayed as green ribbons, and their respective ligands in orange (PDB entry codes: 1SHC and 1IRS). Interacting residues are coloured in red. The insertion/deletion is shown in white. Note that some of the red regions may be included in white ones. B) A section of the dendrogram obtained from the clustering of the PTB domain binding regions (more detailed in figure 4B) is shown for both cases, excluding and including gap regions. Two members of these clusters presenting different (peptide-binding, 1NMB:AB and crystal packing, 1QQG:AB) but overlapping binding regions are highlighted in blue and pink, respectively. A *Si *cut-off of 0.2 (dashed line) is shown for comparison.

Ligands and proteins possess internal degrees of freedom and can adopt various conformational states. Furthermore, many family members often contain sequence inclusions/deletions in loops or C-/N-*termi*, or even additional secondary structure elements, which are often involved in protein interactions. For these reasons, we calculate the *Si *without considering the interacting residues belonging to gap regions in the PSA. This is graphically illustrated in Figure 3A. Two proteins belonging to the same family differ in an insertion of 55 residues, which creates a gap region in the PSA. This additional region is involved in binding and, therefore, increases the number of interacting residues for the protein containing it.

In general, dismissing interacting residues belonging to gap regions in PSAs produces a condensation effect on the clusters at high level of similarity. Additionally, it can also cause reorganization of cluster members at lower levels of similarity. Ignoring gaps for *Si *calculation and applying flexible similarity cut-offs might help in the final clustering and consequent analysis. This is illustrated in Figure 3B, where two contacting domains of the same family presenting two different overlapping binding regions (peptide-binding and crystal packing) are clustered differently depending on considering or excluding gap regions and by applying flexible *Si *cut-offs. As an example, applying a 0.2 cut-*off *when excluding gaps clusters all peptide-binding interfaces separated from the crystal packing interface. This clustering may facilitate further analysis of these different binding regions and their properties.

### Aggregation using the complete-linkage method

Some protein families bind through multiple binding regions with different ranges of residue overlapping. This produces extensions of the binding region definitions and association of two clearly defined regions by a third into a bigger single one. To cope with these usual situations, instead of using the average-linkage used by other authors [10,14], we have rather applied the complete-linkage [9] due to two main properties:

#### Property 1: Complete-linkage is sensitive to zero similarity

**Figure 4:**
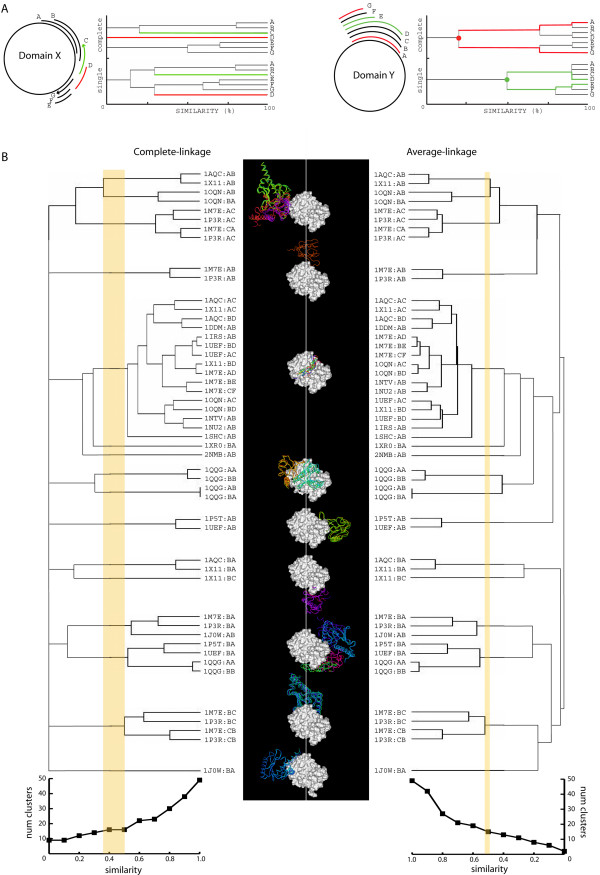
**Aggregation methods for clustering**. A) Schematic comparison between complete- and single-linkage method properties. Two domains (X and Y) with their respective binding regions (A to G) are schematized. Dendrograms obtained from the clustering of PBRs using complete- and single-linkage methods are shown at the right of each domain scheme for comparison. B) Clustering of the PBRs of the PTB domain. The dendrograms derived from the clustering using complete- and average-linkage are shown in the left and right panels, respectively. An example to illustrate the range of similarity that could be used to produce a specific cutting point is represented by the thickness of a yellow line for both methods. The centre of the figure contains the graphical representation of the PTB binding regions obtained using the complete-linkage at zero *Si *cut-off. The PTB domain is represented as a grey surface, and the corresponding binding partners in coloured ribbons. Two graphs representing the number of clusters at different *Si *cut-offs are shown at the bottom.

This method defines at similarity zero all binding regions that do not share interacting residues. Besides, it also assumes that in the same binding region all the members must have some range of similarity among them; otherwise they are split in two separate clusters. This is illustrated in Figure 4A (left panel), where a binding region of domain X might appear as a single one due to the overlapping of several interfaces (A to G). The handling of the three "connector interfaces" (C, D, G) will be responsible of the definition of the final clusters at similarity zero. The clustering is decided based on the higher similarity; C is more similar to B than to D and, on the other hand, G is more similar to F than to D. Therefore, the connectors G and C will be part of the cluster EF and AB respectively, whereas D will belong to a separate cluster. At no similarity, complete-linkage differentiates three binding regions, whereas single-linkage offers only one cluster containing all interfaces. In single-linkage the members having no direct similarity (D, F) are included in the same cluster if there is a "connector interface" (G) having some similarity with both. This enables progressive extensions of a binding region depending on the *Si *cut-off applied. The average-linkage method would have intermediate properties.

#### Property 2: Complete-linkage expands the differences between clusters

Complete-linkage always takes the member with less similarity to join clusters. Domain Y in Figure 4A (right panel) is an illustrative example of binding regions included into others (EFG included in ABCD). The dendrogram shows how the complete-linkage enlarges the differences between both groups more than the single-linkage. The average-linkage would have intermediate values.

These two properties of the complete-linkage method may be very useful for clustering of PBRs. Figure 4B represents a specific example of these properties for all the structurally known binding regions of the PTB (phospho-tyrosine-binding domain) domain (see bellow).

### Threshold values define the final PBRs

The clustering process can be represented by a dendrogram, which shows how the individual objects are successively merged at greater distances into larger and fewer clusters. The branches are proportional in length to the estimated similarity of each binding region with the others. The final clusters depend on the similarity cut-off that is set up.

Binding regions of a family can often present overlapping residues, which makes their definition to be sometimes unclear and arbitrary. Some times there is no unique criteria to adopt in order to define clear PBRs and, in these cases, an appropriate classification may depend on user-based considerations. Illustrative examples are: i) being able to distinguish multi-interfaces *versus *multi-regions (Fig. 1B) in a protein family, ii) distinction of domain-domain *versus *domain-peptide interfaces, and iii) being able to separate and analyze "non-biological" interfaces.

This panorama encouraged us to proceed with the application of several cut-offs within an empirical range of similarities by taking advantage of the clustering properties of the complete-linkage method. The minimum *Si *cut-off value was fixed to zero to give a general view of the binding regions used by a family (property 1). The maximum value was fixed to 0.4 based on our observations (see *Si *cutoff and Definition section). We also pre-calculated the results for 0.1, 0.2, 0.3 *Si *cut-offs to allow flexibility in the analysis of PBRs. Figure 4B shows all the structurally known binding regions of the PTB domain and the clusters for different *Si *cut-offs for complete- and average-linkage. It can be appreciated that the slope is not so drastic in complete – as it is in the average-linkage method. Although offering a similar grouping of elements, the complete-linkage method produces dilatation of the differences among the elements (property 2) and assists in the application of different cut-offs for separation of clusters. As an example, a cut in a specific point (highlighted in yellow bars) gives a wider similarity range for complete – than for average-linkage. The introduced flexibility for choosing cut-offs offers, for example, the possibility to differentiate sub-clusters (*i.e. *2NMB:AB and 1XR0:BA in Figure 4B) and decide to include or exclude them in a specific binding region for comparative analysis.

### Binding regions *vs*. interfaces clustering

In this section we compare SCOWLP with a different method, PRISM [12], to give insights to users into the utilization of our approach and its biological applications compared to other strategies to classify protein interactions. Whereas SCOWLP compares and classifies interfaces based on defined binding regions in the fold of each counterpart (at family level), PRISM compares full interfaces (both partners) in a sequence position independent manner. By using a geometric hashing algorithm it groups interfaces by similarities of the space distribution of interacting residues independently of the fold. Although being two different approaches, both methods can provide a similar number and composition of clusters for a specific protein family; however, differences may also exist in other cases. The following examples are intended to illustrate it (0.2 similarity cut-off used). (1) If a protein family interacts with two different proteins using the same binding region (Single region-multi-interface – Fig. 1B), SCOWLP would always include both interfaces in the same cluster, whereas PRISM would do it only in case it considers similar the distribution of the interfacial residues. This is exemplified in Figure 4B. SCOWLP includes 1j0w:AB in the same binding region cluster as 1m7e:BA and 1p3r:BA, whereas PRISM classifies 1j0w:AB unaccompanied in an only-one-member interface cluster. The same applies to the case of classification of protein-peptide interfaces, where conformational differences of the short peptidic sequences may cause a different PRISM-architecture and, therefore, a separate classification. SCOWLP groups several peptides binding to the same binding region of the PTB domain in one single cluster of 16 members (Figure 4B, cluster 1aqc:AC to 1shc:AB); however, PRISM groups these interfaces in two different clusters of six and seven members. For this specific example, the difference in overall numbers of interfaces is due to the fact that some of the protein-peptide interfaces obtained with SCOWLP are missing in the PRISM clustering (1uef:BD, 1m7e:CF and 1oqn:BD). (2) In the case of structural symmetry (*i.e. *symmetrical protein assemblies and crystal packing), PRISM would include all interfaces in a cluster, whereas SCOWLP would have separated clusters for each binding region. (3) PRISM takes protein chains as a domain unit and therefore does not consider intra-interacting domains, which are considered in SCOWLP.

### Web application

**Figure 5:**
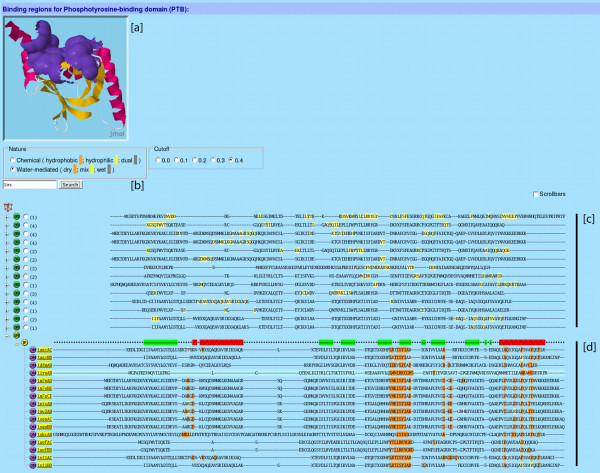
**SCOWLP web application screenshot and utilities**. PTB domain used as an example of the utilities of the SCOWLP database for analysis of PBRs. [a] **3D viewer **allows structural analysis of binding regions the PTB family. [b] The **Manager Box **allows selection of *Si *cut-offs, display of interacting properties and keyword search. [c] Multiple structure alignment of representative PBRs of the PTB domain is provided together with the highlighting of the interacting residues, including solvent-mediated interactions. From the PBR tree, each PBR can be selected to be displayed in the 3D viewer. The PBR tree can be expanded by clicking on its branches to display all interfaces belonging to a particular PBR. [d]. Visualization of the contacting domains. Secondary structure of the domain is displayed, and physico-chemical properties of the interacting residues can be highlighted with the help of the "manager box" (see text for more details about SCOWLP utilities for analysis of PBRs)

We implemented the hierarchical classification of PBRs into the SCOWLP web application. Based on a selected SCOP family, SCOWLP retrieves its binding regions and a summary of the interacting information. The results are generated based on a user-selected similarity cut-off. The analysis of the binding regions can be performed in three different ways (Fig. 5): [a] visualizing the spatial location of each binding region on a representative family structure by using Jmol plug-in [18], [b] keyword search for PDB ids and chains to identify specific complexes, or [c] visualizing the structure-based aligned representative sequences for a binding region with highlighted interacting residues. Once the binding region of interest is localized, a tree-based structure shows three additional classification levels (Fig. 5d): binding region (BR), interface (IF) and contacting domain (DC). All domains in a family that contain interacting information are structurally aligned and their sequences are displayed. Upon selection, the interacting residues can be coloured based on their physico-chemical properties (hydrophobic, hydrophilic or both), and also by the water contribution to the interfacial interactions (dry, wet or dual interaction). A label with the interacting correspondences will appear on each interacting residue when pointed with the mouse. The physico-chemical properties allow the user to distinguish conserved *vs. *variable interactions.

In Figure 5, the PTB domain is used as an example of the utility of the SCOWLP database for analysis of PBRs. In this example, the clustering is selected for similarity cut-off value 0.4 (corresponding dendrogram shown in Figure 4B). A structure-based alignment of the PBRs is obtained, and all interacting residue patterns are highlighted (panel c). A specific binding region is expanded to display all interfaces; in this case corresponding to PTB binding to phospho-tyrosine peptidic ligands. This binding region gets automatically displayed in the 3D viewer for graphical inspection (panel a). This interface is expanded to obtain a structure-based alignment of all PTB domains that use this binding region for recognition. The secondary structure of the domain is displayed at the top of the alignment to help with interpretation of interacting information. The interacting residues are highlighted with different colouring; in this case based on the water contribution to their interfacial interactions (panel d). This information allows comparative analysis of the interfaces, including conservation *vs. *variation of the interactions. In this example we easily are able to analyze (at structure and sequence level) all the interfaces of the PTB domain with different phospho-tyrosine peptides and their interaction patterns. In the example, the three main recognition regions described for the X11 PTB and a peptide motif from the Alzeimer's amyloid precursor protein (APP; PDB entry 1AQC) are displayed and structurally aligned with the recognition regions of other peptides known to bind PTBs in this region. Also, specific differences in the interaction pattern can be further analyzed individually by clicking on each PDB entry code. Analysis of the conservation/variability of the interactions describing an interface may be of great utility for understanding energetic and evolutionary aspects of protein interactions and for helping in rational engineering and design.

## Conclusion

Classification of the regions that a protein family uses to recognize binding partners is important for understanding the interactome. Protein binding regions are often irregular, discontinuous and can share interacting residues among them, making their clustering difficult, and arbitrary. A suitable classification requires proper measurements of similarity between protein binding regions and an appropriate clustering approach. Our approach consists on hierarchical clustering of the PBRs included in the SCOWLP database, which contains detailed interfacial information of proteins, peptides and solvent. We use the complete-linkage method and a similarity index obtained by mapping interacting residues in non-gap regions of pair-wise structural alignments. This approach provides a dilatation of the differences among clusters, making it suitable to isolate poorly separated clusters. In addition, we introduce flexibility in the usage of different similarity cut-offs for PBRs analysis. Our results show that, from 2,561 families containing binding regions, 65% use more than one binding region to interact. Furthermore, from all existing binding regions in SCOWLP, 22% are interacting with more than one protein family. In order to be able to analyze all family binding regions of the PDB in a detailed and comparative fashion we have implemented our PBR classification into the SCOWLP web application.

The current SCOWLP classification and its web application represent a complete framework for the study of protein interfaces and comparative analysis of protein family binding regions. Mining of this information may be of great utility for understanding energetic and evolutionary aspects of protein interactions, reconstruction of protein complexes, understanding protein networks and rational ligand design.

## Availability and requirements

SCOWLP classification of PBRs is freely available at .

## Authors' contributions

JT performed the classification of family binding regions. JT and MP designed the web application. JT and GA performed the PSAs. MTP coordinated and supervised the project. All authors have read and approved the final manuscript.
